# Antisocial Personality Disorder in Bipolar Disorder: A Systematic Review

**DOI:** 10.3390/medicina57020183

**Published:** 2021-02-20

**Authors:** Elvira Anna Carbone, Renato de Filippis, Mariarita Caroleo, Giuseppina Calabrò, Filippo Antonio Staltari, Laura Destefano, Raffaele Gaetano, Luca Steardo, Pasquale De Fazio

**Affiliations:** Department of Health Sciences, University Magna Graecia of Catanzaro, Viale Europa, 88100 Catanzaro, Italy; elvira.carbone@libero.it (E.A.C.); defilippisrenato@gmail.com (R.d.F.); mariaritacaroleo.82@gmail.com (M.C.); giusy878@gmail.com (G.C.); filippostaltari89@gmail.com (F.A.S.); lds@laradestefano.com (L.D.); rafgae74@libero.it (R.G.); staerdo@unicz.it (L.S.J.)

**Keywords:** bipolar disorder (BD), antisocial personality disorder (ASPD), comorbidity, substance abuse, outcome, systematic review

## Abstract

*Background and Objectives*: Bipolar Disorder (BD) is a severe psychiatric disorder that worsens quality of life and functional impairment. Personality disorders (PDs), in particular Cluster B personality, have a high incidence among BD patients and is considered a poor prognostic factor. The study of this co-morbidity represents an important clinical and diagnostic challenge in psychiatry. Particularly, clinical overlap has been shown between antisocial personality disorder (ASPD) and BD that could worsen the course of both disorders. We aimed to detect the frequency of ASPD in bipolar patients with greater accuracy and the impact of ASPD on the clinical course of BD. *Materials and Methods*: A systematic literature search was conducted in PubMed, Embase, MEDLINE and the Cochrane Library through December 2020 without language or time restriction, according to PRISMA statement guidelines. *Results*: Initially, 3203 items were identified. After duplicates or irrelevant paper deletion, 17 studies met the inclusion criteria and were included in this review. ASPD was more frequent among BD patients, especially in BD type I. BD patients with ASPD as a comorbidity seemed to have early onset, higher number and more severe affective episodes, higher levels of aggressive and impulsive behaviors, suicidality and poor clinical outcome. ASPD symptoms in BD seem to be associated with a frequent comorbidity with addictive disorders (cocaine and alcohol) and criminal behaviors, probably due to a shared impulsivity core feature. *Conclusions*: Considering the shared symptoms such as impulsive and dangerous behaviors, in patients with only one disease, misdiagnosis is a common phenomenon due to the overlapping symptoms of ASPD and BD. It may be useful to recognize the co-occurrence of the disorders and better characterize the patient with ASPD and BD evaluating all dysfunctional aspects and their influence on core symptoms.

## 1. Introduction

Bipolar Disorder (BD) is a chronic affective disorder characterized by mood fluctuations with recurrent cycles of mania in BD I, or hypomania in BD II, and depression episodes [1,2], with a highly variable course among patients. The lifetime prevalence of BD is estimated to be around 2–3% in the general population [3] and sub-threshold forms affect at least a further 2% [4]. BD is characterized by a worsening in quality of life [5] and functional impairment [6,7] and is frequently associated with other psychiatric comorbidities that could lead to a worse outcome [8,9,10,11,12,13,14,15]. It has been estimated that patients with BD are exposed to a second psychiatric disorder with longitudinal rates that can be higher than 50% and may reach even 70% [16]. The most common mental disorders that co-occur with BD are drug abuse (33.5%), anxiety disorders (31.8%), alcohol abuse (18.3%), obsessive-compulsive disorder (OCD) (21%), eating disorders (33%), attention deficit hyperactivity disorder (ADHD) (25%), and post-traumatic stress disorder (PTSD) (from 4 to 40%) [17,18,19,20,21,22,23]. In addition, particularly high is the incidences of cluster B personality disorders in BD that are estimated at 41.2% even in the euthymic phase [24] and, represent a poor prognostic factor [25,26,27,28]. The study of this co-morbidity represents an important clinical and diagnostic challenge in psychiatry. Furthermore, Cluster B personality disorders have several common features with BD such as impulsivity, aggressive behavior, and mood instability, enough to induce some authors to consider them part of the bipolar spectrum [29]. Instead, features associated with Cluster B personality disorders may be a dimensional aspect of BD, and when combined could result in greater complexity and severity of the disease [30,31]. Conversely, the presence of BD could worsen the course of a personality disorder [32]. Several studies have shown that the presence of cluster B personality in BD is associated with a higher number of episodes, substance abuse, illegal behavior, a higher rate of suicide risk, and a poor treatment adherence [32,33,34]. In this scenario, researchers have focused on the impact of personality disorder on BD, investigating the common traits that mutually lead to a worse outcome [34]. Particularly, the clinical overlap has been shown between antisocial personality disorder (ASPD) and BD.

According to the DSM-5, the ASPD is described as the existence of constant and pervasive disposition to disregard and disrupt the rights of others. Other specific features include frequent violations of the law, mistreatment of others, deceitfulness, impulsivity, hostility, reckless disregard for the safety of self and others, and imprudent behaviors with lack of guilt, remorse, and empathy [35].

In fact, the prevalence of ASPD can be up to five times higher (4.1%) [36] and appears to be more frequently identified in BD I than BD II [37]. ASPD and BD are both characterized by impulsive behaviors [38,39] and substance use disorder [40,41,42] that frequently lead to trouble with the law [43,44] and suicidal behavior [45,46]. Studies suggested that impulsivity and the frequent abuse of drugs, especially alcohol, cannabis and amphetamines, is associated with sensation seeking and a lack of premeditation in ASPD and that when ASPD is co-morbid with BD it is associated with significant deficits in the ability to delay reward [47,48,49] and greater gray matter volume in the mesolimbic reward system [50]. These characteristics appear more severe when they are combined [50,51,52] and a more in depth characterization of ASPD comorbidity in patients with BD may help clinicians to distinguish both disorders and tailor the treatment. Thus, in an attempt to detect the frequency of ASPD in bipolar patients with greater accuracy and to better clarify the relationship between ASPD and BD, we conducted a systematic review of the existing literature on the frequency of ASPD in BD I, BD II and cyclothymia, according to DSM or ICD diagnostic criteria, evaluating the impact on clinical characteristics and outcomes.

## 2. Materials and Methods

### 2.1. Search Strategy

**Figure 1:**
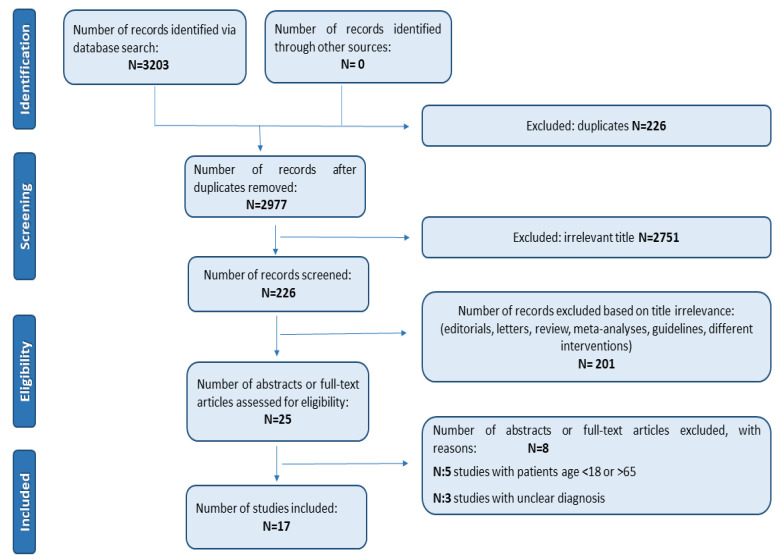
The PRISMA flow chart.

We searched PubMed, Embase, MEDLINE, and the Cochrane Library for articles evaluating the comorbidity between BDs and ASPD published up to 1 December 2020. No language or time restriction were applied. We used the following keywords: *“bipolar disorder OR affective disorder OR mood disorder OR bipolar disorders OR affective disorders OR mood disorders OR BD OR cyclothymia AND antisocial personality disorder OR ASPD OR AABS OR adult antisocial behavior syndrome OR antisocial behavioral syndrome”* sorted by best match. Two researchers independently reviewed all the selected studies. Titles and abstracts of the identified papers were reviewed, and full texts considered relevant were recovered and revised. The reference lists of eligible studies were also hand-screened to search additional and useful studies to be included in the review. To improve the clarity of the review process, the PRISMA Statement criteria and recommendations were followed [53]. Figure 1 shows the research strategy.

### 2.2. Assessment of Study Quality

Quality assessment was conducted using the Mixed Methods Appraisal Tool (MMAT) developed by Pluye et al. [54]. Piloting suggested that the MMAT was a reliable and efficient scoring system for appraising the quality of quantitative, qualitative, and mixed-method studies. It provides a comprehensive manual with detailed instructions. The methodology was evaluated using five criteria: qualitative, randomized controlled, non-randomized, observational descriptive, mixed methods [55]. For each study, a score of 20% was assigned if a criterion was met and 100% if all criteria were met, therefore the total score could range between 20 and 100%. Studies were assigned quality scores by two reviewers (E.A.C. and R.d.F.); scores ranged from 80% to 100%. The quality assessment was finally reviewed and agreed upon the whole review team.

### 2.3. Selection Criteria

Original articles were reviewed and reported. Relevant publications were identified, and the full texts of these articles were retrieved and reviewed. The reference lists of included studies were also screened in order to search useful literature. Studies with patients diagnosed with BD I, BD II or cyclothymia and ASPD according to DSM or ICD criteria, regardless of the phase of the disorder and/or pharmacological treatment, aged 18–65 (we excluded studies on adolescents because personality disorders are not diagnosed in childhood) were included in the review article. Studies with patients diagnosed with BD I, BD II or cyclothymia and ASPD, younger than 18 and older than 65, with neurological comorbidity, or traumatic brain injuries with loss of consciousness were excluded. We considered the studies concerning the lifetime prevalence of ASPD in BD.

### 2.4. Data Collection and Extraction

Two blind researchers (E.A.C. and R.d.F.) independently screened the titles and abstracts of the identified articles and performed data extraction. Articles that met the eligibility criteria were read in the full texts, and in cases of disagreement, such as selection discrepancies, a third researcher (M.C.) made the final decision. Article data included first author name, year of publication, sample size, diagnoses assessed in the study, scales of measurement and statistical data.

## 3. Results

Initially, 3203 items were identified. After deletion of duplicates (226) by two reviewers (E.A.C., R.d.F.), 2977 papers remained. Exclusion of papers by title and abstract was made by two reviewers (E.A.C., R.d.F.) based on assessment of the inclusion and exclusion criteria. This process ended in the exclusion of 2751 papers. The title and abstracts screening was performed for the remaining 226 articles. In all, we excluded 201 articles because they were reviews, meta-analyses, letters to editors, editorials, guidelines, and case reports. Some of them had only a bipolar subgroup or other personality disorder as a comorbidity, or the diagnosis was not clear. Then, 8 manuscripts out of 25 papers were deleted because they did not fulfill the inclusion criteria: 5 papers included patients aged <18 or >65, and 3 papers had unclear diagnosis focusing on personality disorders and not specifying the diagnosis. The remaining 17 papers were deemed eligible and included in the present review (Table 1). A great heterogeneity was reported among studies included and wide variability in the sample number (from *N* = 21 to *N* = 43,093) [49,56]. ASPD prevalence in BD ranged between 4.8% and 63% [47,57] and was higher in BD I [37] than II [17,49,58] and, in particular, in BD patients with substance use disorder (SUD) comorbid [47,48,49] with combined cocaine and alcohol abuse was most frequent [48,49,59]. Patients with BD and ASPD in comorbidity showed early onset [58], a higher number of depressive and manic episodes [47], higher scores of depression [59] and psychosis [47], more aggressive [60], and impulsive [47,61] traits and more suicide attempts [47]. The psychometric tools used to assess the psychopathology were the Alcohol Use Disorder and Associated Disabilities Interview Schedule (AUDADIS) [49,57], the 12-Item Short Form Survey (SF-12) [49,57], the Barratt Impulsiveness Scale (BIS-11) [47,58], the Temperament Evaluation of Memphis, Pisa, Paris and San Diego-auto-questionnaire version (TEMPS-A) [62], Hamilton Depression Rating Scale (HDRS) [60,63], Young mania rating scale (YMRS) [60,63], Brown–Goodwin Aggression Scale (BGA) [60], and the Schedule for Affective Disorders and Schizophrenia (SADS-C) [47,63].

## 4. Discussion

To the authors’ knowledge, this is the first systematic review that assessed the association and the impact of ASPD and BD. It is worth mentioning how scarce the studies are that evaluate this association despite the high clinical relevance. The literature demonstrated a high incidence of Axis II personality disorders in patients with DB [24,68] and the more frequent were Cluster B personality disorders [19,25,26,27,69] followed by C and A [17,47,58,67]. Antisocial personality disorders [59] together with narcissistic [19,70] and histrionic personality disorder were diagnosed most frequently in BD patients, even in recent-onset BD patients [56]. Pica et al., found that 62% of BD patients had PDs and ASPD was present in 15–39% [67]. Moreover, as can be seen from the studies, a prevalence of 30% ASPD in BD was reported, with a superior incidence in BD I than in BD II. A greater occurrence of antisocial behavior [29] and greater impulsivity during episodes, especially during mania has been demonstrated [52]. The clinical severity due to the co-occurrence of the two disorders and therefore the greater demand for access to clinical services could explain this higher prevalence. This great variability found in this review (4.8% to 63% [47,57]) may be due to the variable sample size and population selected of included studies (from *N* = 21 to *N* = 43,093) [49,56], the retrospective nature of the included studies, the methodological differences (e.g., instruments used, phase of the disorder at the time collection) that may negatively affect the ability to discriminate between ASPD and BD diagnoses due to the high prevalence of BD in the general population and the lack of a precise tool for ASPD diagnostic assessment.

As expected, ASPD in comorbidity with BD is associated with a more severe course of illness and poor responsiveness and adherence to treatment [71,72]. ASPD in BD patients was associated with a poor outcome after a manic episode [73], a higher rate of suicide attempts [63,74], and a worse course of illness [75], with greater service admission [76]. Moreover, the greatest number of depressive events associated with the comorbidity with PDs results in a reduction in the quality of life, and since this is the most frequent suicide attempt during depressive phases, this may explain why patients with comorbidity also have a greater tendency to attempt suicide [77]. Patients with BD and ASPD showed an earlier onset of affective symptomatology [58], higher psychopathological burden [58,63], and a higher number of affective episodes [47]. An earlier age at onset of the antisocial behavior has been also described [32] that often persists in adulthood [78]. Notably, earlier onset is associated with a poorer prognosis in both disorders [79,80], probably due not only to the genetic contribution [81,82] but also to the environmental influences (e.g., child maltreatment, abuse, violence, harsh and inconsistent parental discipline, and lower quality caregiving) [83,84]. These patients have been also found to have a higher score of aggressivity measured with the BGA [60], higher impulsivity measured by the BIS-11 [47,58,61], and more suicide attempts [47]. Manic episodes can more easily lead to criminal penalties, illegal conduct therefore indirectly impacts treatment adherence [85]. Low treatment rates were found in patients with ASPD [57] and reflect affected individuals’ lack of insight into the seriousness of their problem and consequently lack of effective interventions. Even if BD demonstrated a higher rate of treatment compared to patients affected by ASPD, the co-occurrence of two disorders, often in younger patients, may interfere with treatment, as well as the ability to adhere to the treatment, with consequent poor outcome [59].

ASPD symptoms were associated with a history of alcohol or other SUDs as well as smoking. Substance abuse before 15 years is strongly related to ASPD [64,86] and ASPD symptoms were related to age at BD onset independently of gender [58]. Lev-Ran and colleagues estimated the 12-month prevalence of BD, SUD (cannabis), and ASPD as 49.9%, compared to 18.2% of patients with BD and without SUD [49]. Studies described cocaine and alcohol combined abuse as most frequent in BD and ASPD [48,49]. A more severe course of illness was found in comorbid SUD in bipolar patients, including an earlier onset, more rapid progression to dependence, and greater social, legal, and physical use consequences [64]. The frequent comorbidity with addictive disorders, suicidality, and criminal behaviors described, could be probably due to a shared impulsivity core feature [52]. A high level of impulsivity has been shown in patients affected by BD in comorbidity with ASPD measured by the Immediate Memory Task (IMT) and Two Choice Impulsivity Paradigm (TCIP). Moreover, faster Immediate Memory Task (IMT) reaction times in BD combined with SUD compared to BD alone have been reported [52]. The results suggest that loss of compensatory mechanisms may lead to more severe impulsivity in the combined disorders [52]. Another important aspect is the influence of temperament, which, in BD patients, can give rise to the predisposition to develop ASPD. Perugi et al. evaluated the influence of the affective temperament and psychopathological traits in a sample of patients with BD I and ASPD and found a higher incidence of hyperthymic temperaments in this population (8.49%) [62], suggesting that affective temperament influences clinical features of BD when in comorbidity with Axis II disorders [62].

**Figure 2:**
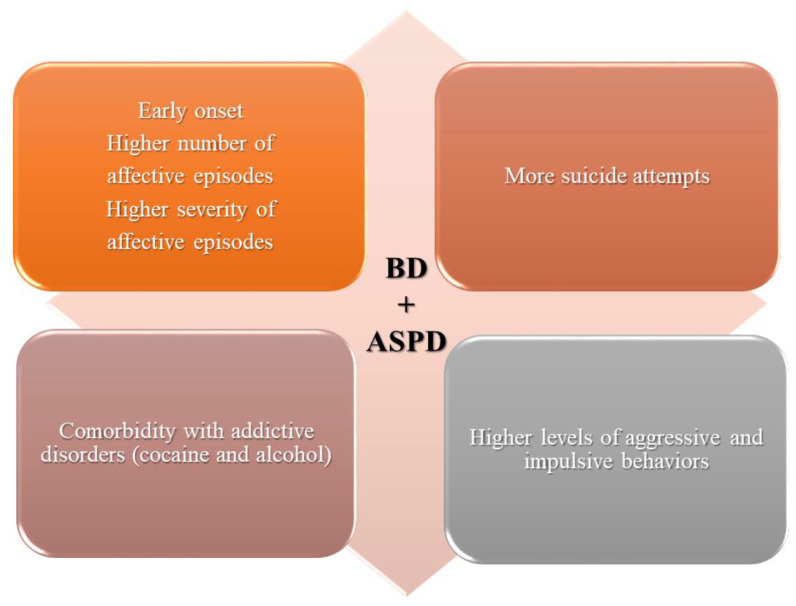
Main results.

**Figure 3:**
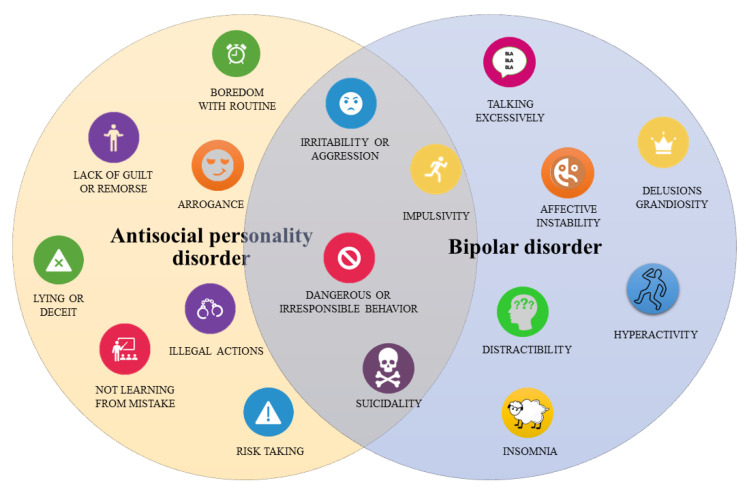
Overlapping symptoms between ASPD and BD.

Thus, comorbidity with ASPD seems to impact not only the onset but also the cyclical nature of BD, increasing the number of episodes, psychopathological scores, suicide attempts, and poor adherence to treatment (Figure 2 and Figure 3). Considering the shared symptoms such as impulsive and dangerous behaviors (i.e., substance abuse, driving recklessly, inappropriate sexual behavior), in patients with only one disease, misdiagnosis is a common phenomenon due to the overlapping symptoms of ASPD and BD. It may be useful to recognize the co-occurrence of the disorders and disentangle whether the two disorders are independent or interdependent conditions.

### Limits and Future Directions

This review presents gray areas that deserve to be further explored. Potential limitations to consider include: (1) studies selected often determine the course of illness retrospectively. Moreover, many studies enrolled patients regardless of the phase of the illness. The results of the diagnostic assessments may be affected by the state of illness, thus reducing the quality of the included studies; (2) great heterogeneity and wide variability in the sample number and population selected was reported among studies making it difficult to correctly define the prevalence; (3) mechanisms underlying ASPD or AABS characteristics may be different in individuals with BD compared to those without BD; (4) additional comorbidities with further personality disorders were not systematically evaluated by the included studies. Although this does not affect the epidemiological results, it could limit their psychopathological interpretation. It should be necessary to better characterize comorbidity, evaluating all dysfunctional aspects of diseases and how they could influence core symptoms and comorbidity. It may be necessary to evaluate psychopathy within BD, as it is plausible that comorbid antisocial traits are different from the psycho-antisocial traits. It should also be necessary to evaluate whether patient profiles with comorbidity could benefit from different treatments. Psychopathy has not been investigated because of the difficulty of its classification according to DSM in relation to ASPD.

## 5. Conclusions

ASPD was estimated as more frequent among BD patients, especially in BD type I. BD patients with ASPD as a comorbidity seemed to have early onset, a higher number of manic and depressive episodes, more severe affective episodes, higher levels of aggressive and impulsive behaviors. Comorbidity is associated with a worse prognosis, increased frequency of relapse, poor clinical outcome, higher frequency of dangerous behaviors, a higher rate of suicide attempts and poorer treatment adherence. Furthermore, ASPD symptoms in BD patients seem to be also associated with frequent comorbidity with addictive disorders (alcohol or cocaine abuse disorder), suicidality, and criminal behaviors, probably due to a shared impulsivity core feature. Therefore, we suggest better characterization of the patient with BD and ASPD. Based on the literature data, considering the comorbidity between BD and ASPD and common elements between ASPD and psychopathy, it would be desirable to carry out clinical trials that also investigate in-depth the comorbidity among the three conditions together. Indeed, the presence of psychopathy in patients with BD and ASPD may have important consequences in clinical, prognostic, and therapeutic terms.

## Figures and Tables

**Table 1:** The main characteristics of the included studies.

Authors, Years	Sample	Measurements	Results	Comments	MMAT
Goldstein et al., 2017 [57]	*N* = 36,309	AUDADIS-5; SF-12	Lifetime prevalence: ASPD + BD1: 11.8% AABS + BD1: 4.8% OR (C.I.) lifetime of BD 1 comorbidity: ASPD 2.9 (1.93–4.28) AABS 1.9 (1.43–2.50)	Comorbidity is higher in the ASPD than in the AABS. Patients with ASPD has a 3 times fold risk of BD, while it is 2 times greater if it has AABS	*****
Lev-Ran et al., 2013 [49]	*N* = 43,093	AUDADIS-IV; SF-12	12-month prevalence: BD + CUD + ASPD: 49.9% BD + No CUD + ASPD: 18.2% OR (C.I.): 2.75 (1.63–4.64)	The bipolar patient with CUD is almost 3 times more likely to have ASPD than the bipolar patient without CUD	*****
Swann et al., 2013 [58]	*N* = 55 4 BD II 51 BD I 14 = no Axis II disorder (8 men and 6 women) 35 ASPD (20 men and 15 women) 23 Borderline 8 men and 15 women 17 (7 men and 10 women) both disorders	Diagnosis was made by Structured Clinical Interview for DSM-IV SCID-II Schedule for Affective Disorders and Schizophrenia (SADS-C) Barratt Impulsiveness Scale (BIS-11)	Prevalence: 35 ASPD of 55 BD:63% 17 ASPD + Borderline of 55: 30.9%. Number of episodes: ASPD symptoms predicted a history of many depressive and manic episodes (but not either type alone) and a early onset. BIS-11 score: Total, motor, and attentional BIS-11 scores were predicted significantly by borderline symptom scores with no significant contribution from ASPD scores. Suicide attempts: Impulsivity in ASPD + BD did not contribute significantly to history of suicide attempt SUD Comorbidity: ASPD symptoms predicted history of alcohol, other substance-abuse disorder, and smoking.	ASPD symptoms were more strongly related to course of illness (i.e., early age at onset, frequency of affective episodes, suicide attempts and substance-related disorders) but not to impulsivity.	****
Mueser et al., 2012 [59]	*N* = 103	SCID-II; Time-line Follow-back Calendar; AUS; DUS; SATS; BPRS; GAS; Knowledge Test; SPSI; FAS; SF-12; FEIS	Prevalence: 21 ASPD of which 11 BD: 52%	Over half of the antisocial patients are also bipolar	*****
Perugi et al., 2012 [62]	*N* = 106	CGI-BP; TEMPS-A; SAS; IPSM; SIMD-R	Prevalence: BD I + ASPD: 8.49%	The authors evaluated the impact of the affective phase of BD1 on axis II diagnosis, concluding that ASPD is more represented among hyperthymic than cyclothymic, depressive or euthymic patients and affective temperaments may influence both clinical features and axis I and II comorbidities.	****
Swann et al., 2011 [61]	*N* = 133 46 HC 21 BD without personality disorders 50ASPD without BD 16BD + ASPD	Immediate Memory Task (IMT) Two Choice Impulsivity Paradigm (TCIP)	Prevalence: 16 ASPD of 37 BD: 43.24% Impulsivity: Impulsivity was increased in the combined disorders compared to both disorders alone. Outcome: In combined ASPD and BD increased reaction speed, impulsive response bias, and reward- delay impulsivity occurred. It was independent of substance-use disorder history.	The combination of ASPD and BD was associated with more impulsive TCIP performance compared to HC. Compensatory mechanisms for impulsivity in uncomplicated ASPD or BD appear to be compromised or lost when the disorders are in comorbidity.	****
Goldstein et al., 2010 [17]	*N* = 2442	AUDADIS-IV	Prevalence: BD1 + ASPD: 45.1% (*p* < 0.0001) BD2 + ASPD: 8.2% (*p* < 0.0122)BD1 + AABS: 32.4% BD2 + AABS: 5.8%	In subjects with PTSD and ASPD, comorbidity with DB 1 is the strongest evidence, that with DB 2, although lower, however, is statistically significant.	*****
Swann et al., 2010 [47]	*N* = 197 78 HC 34 ASPD 61 BD 24 BD + ASPD	SCID-II; SADS-C; BIS-11	Prevalence: 12.4% of total sampleSADS-C score: Higher scores of depression and psychosis BIS-11 score: higher subscale and total score Suicide attempts: BD + ASPD: 65.4% BD: 34.9%SUD Comorbidity: BD + ASPD: 91.3% BD: 66.7%Number of episodes: Higher number of manic and depressive episodes	Comorbidity is associated with a greater tendency to depression and psychosis, an increased number of depressive and manic episodes, greater impulsivity, greater risk of SUD and suicide.	****
Garno et al., 2008 [60]	*N* = 100 73 BD1 27 BD2	SCID-I; SCID-II; HDRS; YMRS; CTQ; BGA	Prevalence DB + ASPD: 6.25% Comorbidity and BGA: Higher BGA total score (*p* 0.008)	Bipolar patients with ASPD have more aggressive traits	****
Mitchell et al., 2007 [48]	*N* = 166	MINI	Prevalence ASPD in BD + SUD%; OR (C.I.): BD + COCA: 52.8%; 1.86 (0.81–4.26) BD + COCA + ALCOL:60%; 2.50 (1.23–5.08)	ASPD is more associated with the bipolar group with cocaine dependence (almost twice the risk) or cocaine plus alcohol (twice and a half risk)	****
Maina et al., 2007 [19]	*N* = 204 BD = 21 BD I = 4; BD II = 17 BD + ASPD = 6	Yale-Brown Obsessive-Compulsive Scale (Y-BOCS); SCID-I; SCID-II;	Prevalence DB + ASPD: 6% SUD Comorbidity: SUD + BD: 28.6%. Comorbidity: prevalence of antisocial personality disorders + BD: 28.6%	Clinically relevant effects of comorbid BD on the personality features of OCD patients. A higher rate of narcissistic and ASPD in BD/OCD patients.	****
Mueser et al., 2006 [64]	*N* = 178	SCID-I; SCID- II; BPRS; GAS; TLFB; ASI; AUS; DUS; SATS	Prevalence BD + AABS: 21.2% Prevalence BD + ASPD: 21.1%	The prevalence of AABS and ASPD in the DB is superimposable	****
Garno et al., 2005 [63]	*N* = 100 73 BD1 27 BD2	SCID-I; SCID-II; CTQ; YMRS; HAM-D; SADS-C	Prevalence BD + ASPD: 6%	There is no statistically significant correlation between YMRS and HAM-D scores with ASPD.	****
Mueser et al., 1999 [65]	No ASPD/CD = 293 CD Only = 293 Adult ASPD Only = 293 Full ASPD = 293 Schizophrenia (28%), schizoaffective disorder (24%), bipolar disorder (22%), major depression. (19%) and other (7%)	SCID; MMS; CRS; MAST; DAST; CAGE; TACE; TWEAK; ACI; AUDIT.	Prevalence BD + Adult ASPD Only: 24% Prevalence BD + Full ASPD: 22% SUD Comorbidity: Full ASPD group had the highest rate of substance use disorder, followed by either the CD Only or Adult ASPD Only groups, with the No ASPD/CD group lowest	Childhood CD and adult ASPD represent independently significant risk factors for substance use disorders in patients with schizophrenia- spectrum and major affective disorders.	****
Jackson & Pica, 1993 [66]	112 psychiatric inpatients 11 antisocial personality disorder, 65 had other forms of personality disorders, 36 no personality disorder. 35 recent-onset schizophrenic patients (27 men, 6 women), 26 recent-onset bipolar disorder patients (14 men, 12 women), 30 unipolar affective disorder patients (14 men, 16 women), and 21 (11 men, 10 women) with mixed disorders (e.g., anorexia nervosa, substance abuse, somatoform disorders)	SCID, Royal Park Multidiagnostic Instrument for Psychoses, SAPS, SANS, BDI BRMS	Prevalence: 4 patients of 11 antisocial are affected by BD.	Patients with ASPD were younger, with lower level of education and higher levels of many dysfunctional behaviors, as delinquency, sexual intercourse, drink/drugs abuse, thefts, vandalism, inconsistent work, irritability/aggressive, impulsivity, recklessness, continual antisocial behavior than patients with other or none PDs.	****
Turley et al., 1992 [56]	21 recent onset BD (12 man and 9 women)	MCMI-II); SIDP; BDI; SAPS; BRMS; SCID-P	The overall ratio of personality disorders identified was virtually equivalent for the MCMI- I1 and the SIDP. However, the MCMI-I1 was far more likely to make multiple diagnoses than the SIDP. The MCMI-I1 identified a total of 52 personality disorders compared with 30 for the SIDP.	Narcissistic and Antisocial personality disorders were the most prevalent disorders in this sample of Bipolar disordered patients, followed by Histrionic and Passive-Aggressive disorders	****
Pica et al., 1990 [67]	*N* = 26 16 BD 10 Schizoaffective Disorder	SIDP; SCID-P; RPMIP; BDI; BRMS; SAPS; SANS	Prevalence BD + ASPD: 15.39%	Patients with BD showed a high frequency of PDs.	****

MMAT scores: ***** 100%; **** 80%; *** 60%; ** 40%; * 20%. AABS: Adult Antisocial behavioral syndrome; ACI: Alcohol Clinical Index; ASI: Addiction Severity Index; ASPD: Antisocial personality disorder; AUDADIS: Alcohol Use Disorder and Associated Disabilities Interview Schedule; AUDIT: Alcohol Use Disorder Identification Test- Clinical Procedure; AUS: Alcol Use Scale; BD: Bipolar Disorder; BDI: Beck Depression Inventory; BGAS: Brown-Goodwin Aggression Scale; BIS-11: Barratt Impulsivity Scale; BPRS: Brief Psychiatric Rating Scale; BRMS: Bech-Rafaelsen Mania Scale; CAGE: Cut Down on Drinking, Annoyed, Guilt and Eye-opener Test; CGI-BP: Clinical Global Impression-BP; CTQ: Childhood Trauma Questionnaire; C.I.: Confidence Interval; CRS: Clinician Rating Scales; CUD: Cannabis Use Disorder; DAST: Drug Abuse Screening Test; DUS: Drugs Use Scale; FAS: Family Attitude Scale; FEIS: Family Experiences Interview Schedule; GAS: Global Assessment Scale; HC: Healthy Control; HDRS: Hamilton. Depression Rating Scale; IMT: Immediate Memory Task; IPSM: Interpersonal Sensitivity Measure; MAST: Michigan Alcohol Screening Test; MCMI-II: Millon Clinical Multiaxial Inventory; MINI: Mini International Neuropsychiatric Interview; MMAT: mixed-method appraisal tool; MMS: Mini- Mental State; OCD: obsessive-compulsive disorder; OR: Odd Ratio; OR: adjusted Odd Ratio; PTSD: Post-Traumatic Stress Disorder; RPMIP: Royal Park Multi- Diagnostic Instrument for Psychosis; SADS: Schedule for Affective Disorders and Schizophrenia; SANS: Scale for the Assessment of Negative Symptoms; SAPS: Scale for the Assessment of Positive Symptoms; SASI: Separation Anxiety Symptom Inventory; SATS: Substance Abuse Treatment Scale; SCID- I:Structured Clinical Interview for DSM-IV Axis I Disorders; SCID-P: Structured Clinical Interview for Personality Disorders; SCID-II: Structured Clinical Interview for DSM-IV Axis II Disorders;SF-12: 12-Item Short Form Health Survey; SIDP: Structured Interview for DSM-III personality; SIMD-R: Semi-structured interview for Mood Disorder; SPSI: Social Problem Solving Inventory; SUD: Substances Use Disorder; TACE: Tolerance, Annoyed, Cut Down, and Eye- opener Test; TCIP: Two Choice Impulsivity Paradigm; TEMPS-A: Temperament Evaluation of Memphis, Pisa, Paris and San Diego scale; TLFB: Time-line Follow-back; TWEAK: Tolerance, Worry, Eye-opener, Amnesia and Cut Down on drinking Test; YMRS: Young Mania Rating Scale.

## Data Availability

Data available on request.
